# Targeting NF-κB pathway for the therapy of diseases: mechanism and clinical study

**DOI:** 10.1038/s41392-020-00312-6

**Published:** 2020-09-21

**Authors:** Hui Yu, Liangbin Lin, Zhiqiang Zhang, Huiyuan Zhang, Hongbo Hu

**Affiliations:** 1 Department of Rheumatology and Immunology, National Clinical Research Center for Geriatrics, State Key Laboratory of Biotherapy, West China Hospital, and West China School of Basic Medical Sciences & Forensic Medicine, Sichuan University, and Collaborative Innovation Center for Biotherapy, Chengdu, China; 2 Immunobiology and Transplant Science Center, Houston Methodist Hospital, Houston, TX 77030 USA

**Keywords:** Molecular medicine, Translational immunology

## Abstract

NF-κB pathway consists of canonical and non-canonical pathways. The canonical NF-κB is activated by various stimuli, transducing a quick but transient transcriptional activity, to regulate the expression of various proinflammatory genes and also serve as the critical mediator for inflammatory response. Meanwhile, the activation of the non-canonical NF-κB pathway occurs through a handful of TNF receptor superfamily members. Since the activation of this pathway involves protein synthesis, the kinetics of non-canonical NF-κB activation is slow but persistent, in concordance with its biological functions in the development of immune cell and lymphoid organ, immune homeostasis and immune response. The activation of the canonical and non-canonical NF-κB pathway is tightly controlled, highlighting the vital roles of ubiquitination in these pathways. Emerging studies indicate that dysregulated NF-κB activity causes inflammation-related diseases as well as cancers, and NF-κB has been long proposed as the potential target for therapy of diseases. This review attempts to summarize our current knowledge and updates on the mechanisms of NF-κB pathway regulation and the potential therapeutic application of inhibition of NF-κB signaling in cancer and inflammatory diseases.

## Introduction

NF-κB forms a family of transcription factors that play essential roles in multiple physiological and pathological processes. There are two different NF-κB pathways, the canonical and non-canonical NF-κB pathways, with different activating mechanisms.^1–4^ It is well established that the canonical NF-κB is activated to respond to a diversity of external stimuli involved in inflammation, immune response, cell proliferation, differentiation, and survival.^4–7^ The crucial step of activation of the canonical NF-κB is phosphorylation-dependent activation of the IKKs (IκB kinases) complex.^7,8^ Consequently, the inhibitory IκB proteins are phosphorylated and subjected to the ubiquitination-dependent degradation by proteasome, liberating the κB transcription factor to translocate to nucleus and activate the target genes. The activation is quick but transient, since NF-κB also induces expression of the negative regulators like IκBα, A20, and p105, forming a negative feedback mechanism.^6,9,10^ On the other hand, the non-canonical NF-κB is activated only through a handful of TNF superfamily receptors, indicating that the biological functions of this branch of pathway are more specific.^1–3,11^ NIK (NF-κB-inducing kinase), the key kinase in this pathway, remains below the detectable level in the steady-state condition due to the TRAF3 (TNFR-associated factor 3)-dependent ubiquitination-mediated degradation.^12,13^ Upon stimulation, TRAF3 is degraded by E3 ubiquitin ligase cIAP (cellular inhibitor of apoptosis), leading to NIK accumulation.^14^ Consequently, NIK, together with IKKα, phosphorylates p100, which is further processed to p52, releasing RelB/p52 dimer to translocate into nuclear for target gene activation.^13,15,16^ Non-canonical NF-κB pathway is responsible for the development of immune cells in multiple layers. For instance, this pathway is required for maturation and function of TECs (thymus epithelium cells), which are essential for T-cell development in the thymus.^17–19^ This pathway is also recognized as a critical regulator in the development of SLO (secondary lymphoid organ).^3,20,21^ While studies also illuminate the importance of this pathway in the development of TLO (tertiary lymphoid organ) and chronic inflammatory diseases.^3,20–25^ In this review, we will discuss regulation of NF-κB pathway and how this pathway is involved in immune response and as the potential target for inflammation-related disease and cancers.

## Activation of the canonical NF-κB pathway

**Fig. 1:**
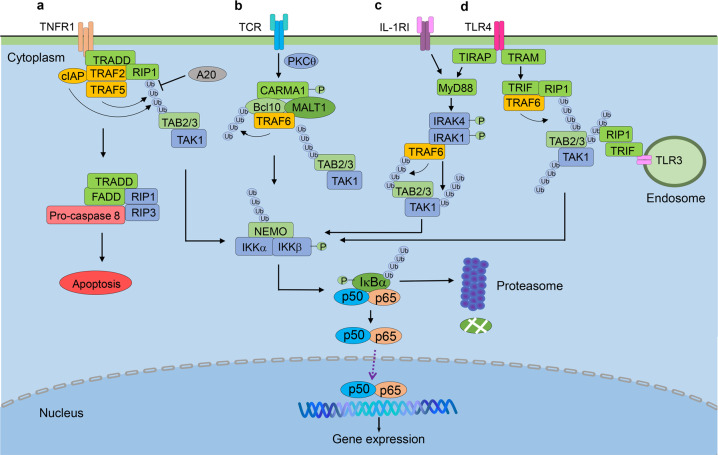
Activation and regulation of canonical NF-κB pathway. **a** Ligation of TNFR leads to the recruitment of TRADD and interaction of the E3 ubiquitin ligases cIAP1/2, TRAF2 with the protein kinase RIP1. RIP1 then is K63-ubiquitinated and recruited to NEMO, resulting in the formation of the TAK1-IKK complex. TAK1 phosphorylates and activates IKKβ that in turn induces the phosphorylation and degradation of IκBα, allowing NF-κB dimers to translocate to the nucleus and drive the transcription of the target gene. **b** TCR stimulation induces canonical NF-κB activation through the CARD11/Bcl10/MALT1 (CBM) complex. Upon stimulation, CARD11 (CARMA1) is recruited and phosphorylated by PKC-θ, leading to the recruitment of Bcl10 and MALT1 to form the CBM complex. MALT1 then recruits TRAF6, which mediates K63 ubiquitination of itself and Bcl10, followed by the activation of TAK1 and IKK-mediated canonical NF-κB activation. **c**, **d** TLR and IL-1RI mediate signal transduction through MyD88. TLR4 recruits TIRAP and TRAM, which recruits MyD88 and TRIF, respectively, and IL-1RI recruits MyD88 directly. MyD88 induces the recruitment of IRAK1 and IRAK4, which further recruit TRAF6 to activate TAK complex and downstream signaling pathways. The activity of canonical NF-κB is regulated at multiple levels. The expression of A20 is activated by NF-κB, which deubiquitinates RIP1, TRAF6 and NEMO to destabilize the IKK complex. TRADD, TNF-R-associated death domain; RIP1, receptor-interacting protein 1; cIAP1/2, cellular inhibitor of apoptosis 1 and 2; TRAF2/5, TNF-R-associated factor 2 and 5; NEMO, NF-κB essential modulator; TAK1, TGF-β-activated protein kinase 1; IKKβ, inhibitor of κB(IκB) kinase β; TCR, T cell receptor; PKC-θ, protein kinase C-θ; TLR4, Toll-like receptor 4; TIRAP, Toll/IL-1 receptor adaptor protein; TRAM, TRIF-related adaptor molecule; MyD88, myeloid differentiation primary response gene 88; TRIF, TIR domain-containing adaptor inducing interferon beta; IRAK-1/4, IL-1 receptor-associated kinase 1 and 4

The NF-κB family has five members, p65 (RelA), RelB, c-Rel, p105/p50, and p100/p52, all of which share a common amino-terminal REL homology domain, RHD.^6,7,26^ It is well established that RelA and p50 heterodimers are responsible for transcription of target genes when the canonical NF-κB pathway is activated, while RelB and p52 form a heterodimer in non-canonical NF-κB pathway.^3,27^ In the steady-state settings, RelA and p50 are sequestered in the cytoplasm by the IκB (inhibitor of NF-κB) proteins, which consist of three groups: the typical IκB proteins (IκBα, IκBβ, and IκBε),^28–30^ the precursor proteins (p100 and p105),^31^ and the atypical IκB proteins (IκBζ, BCL-3 and IκBNS).^29,32,33^ The central event in canonical NF-κB activation is the signal-induced phosphorylation of IκB molecules by IKKs. IKK consists of two homologous catalytic subunits IKKα (also known as IKK1) and IKKβ (also known as IKK2), and a regulatory subunit IKKγ (also known as NF-κB essential modulator, NEMO).^34^ IKKβ is essential for canonical NF-κB activation in response to proinflammatory cytokines and various microbial products, while IKKα mainly regulates non-canonical NF-κB activation.^8,9,35^ NEMO lacks catalytic function, and is required for canonical NF-κB activation. The N-terminal of NEMO binds to IKKs, while the C-terminal of NEMO mediates its interaction with upstream signaling adapters. IKKβ kinase activity depends on the oligomerization of IKKα/β/γ.^8,10,34^ Activated IKKβ induces the phosphorylation of IκB protein, leading to the K48-linked ubiquitination of IκBs and their subsequent degradation (Fig. 1), which results in release of NF-κB dimers from cytoplasmic inhibition.^7,26^ Released NF-κB dimers translocate to the nucleus and drive transcription of target gene.^6,7,32^ Canonical NF-κB is rapidly activated in both innate and adaptive immune cells by numerous signals through innate PRRs (pattern-recognition receptors), TCR (T-cell receptor), BCR (B-cell receptor), proinflammatory cytokine receptors and etc (Fig. 1).^5,6,27^ Specific adaptor molecules, ubiquitin ligases, and protein kinases are involved in the various pathways to activate IKK complex.^31,34^

### Activation of canonical NF-κB in the innate immune response

PRRs expressed on innate immune cells, such as macrophages, monocytes, neutrophils, and DCs (dendritic cells), recognize microbial PAMPs (pathogen-associated molecular patterns) or DAMPs (damage-associated molecular patterns) released by damaged tissue, to induce the expression of proinflammatory cytokines such as TNF-α (tumor necrosis factor-alpha), IL-1 (interleukin-1), IL-6, IFN-I (type I interferons), chemokines, and anti-microbial proteins,^36–39^ leading to the inflammatory response to eliminate pathogens and repair the damaged tissues. There are five PRR families identified in mammalian, including TLRs (Toll-like receptors), NLRs (NOD-like receptors), RLRs (RIG-I-like receptors), CLRs (C-type lectin receptors), and cytosolic DNA sensors.^36,40,41^

TLRs are composed of N-terminal LRRs (leucine-rich repeats) for ligand recognition, a transmembrane region, and cytosolic TIR (Toll-IL-1 receptor) domain that mediates downstream signal transduction.^37,42^ They are the most well-characterized PRRs. TLR1, TLR2, TLR4, TLR5, and TLR6 are localized on the plasma membrane and mainly recognize microbial components. TLR3, TLR7, TLR8, and TLR9 are presented in intracellular vesicles such as ER (endoplasmic reticulum) and endosomes, sensing different types of nucleic acids.^42,43^ Upon LPS stimulation, for example, TLR4 recruits TIRAP (Toll/interleukin-1 (IL-1) receptor adaptor protein) and TRAM (TRIF-related adaptor molecule, also known as TICAM2). TIRAP and TRAM recruits MyD88 (myeloid differentiation primary response gene 88) and TRIF (TIR domain-containing adaptor inducing interferon beta, also known as TICAM1), respectively, for downstream signaling.^40,42,44^ Signals from MyD88 lead to the recruitment of IRAK1 and IRAK4 (IL-1 receptor-associated kinase 1 and 4), TRAF6 and TAK1(TGF-β-activated kinase 1).^45,46^ TRAF6 acts as an E3 ubiquitin ligase to mediate autoubiquitination, which further forms a complex with TAB2 (TAK1 binding protein 2), TAB3 and TAK1, leading to auto-phosphorylation and activation of TAK1.^47^ Subsequently, TAK1 mediates the phosphorylation and activation of IKKs, as well as downstream canonical NF-κB pathway, resulting in the expression of proinflammatory cytokines.^5,48^ TRIF-dependent signaling activates both canonical NF-κB and IRF3 (interferon regulatory factor 3) to induce the production of proinflammatory cytokines and IFN-Is. In brief, TRIF recruits TRAF6 and RIP1 (receptor-interacting protein 1) to activate canonical NF-κB, and TRAF3 is required for IRF3 activation, respectively^37,45,49^ (Fig. 1). Similarly, TLR3 recognizes dsRNA and activates canonical NF-κB in TRIF-dependent way.^50^ TLR2 forms a heterodimer with either TLR1 or TLR6 and recognizes components of bacteria, mycoplasma, and viruses.^51,52^ TLR1/TLR2 and TLR6/TLR2 heterodimers activate canonical NF-κB in TIRAP- and MyD88-dependent ways.^39,53^ TLR7 and TLR9 are located in the ER membrane and exclusively expressed in pDCs (plasmacytoid DCs), which produce massive IFN-I during viral infection,^54,55^ they also induce activation of canonical NF-κB in MyD88-dependent manner after sensing ssRNA and CpG DNA, respectively.^56,57^

The RLR family includes RIG-I (retinoic acid-inducible gene I), MDA5 (melanoma differentiation-associated gene 5), and LGP2 (laboratory of genetics and physiology 2 and a homolog of mouse D11lgp2).^58,59^ RIG-I recognizes RNA viruses by sensing short dsRNA with 5′ triphosphate ends,^60–62^ and MDA5 prefers to recognize long dsRNA,^63,64^ while LGP2 sequesters dsRNA and thereby functions as a negative regulator of RLR signaling.^65–68^ RIG-1 and MDA5 share N-terminal CARDs (caspase activation and recruitment domains), central DExD/H box helicase/ATPase domain, and C-terminal regulatory domain.^58,69,70^ Upon activation, RIG-I and MDA5 interact with IPS-1 (interferon beta promoter stimulator protein 1, also known as MAVS, VISA) through CARD domain.^71–74^ IPS-1 forms signaling complexes with multiple proteins and mediates downstream signaling that drives the production of IFN-I and canonical NF-κB-dependent cytokines.^69,75^ FADD (Fas-associated death domain) and RIP1, known as mediators of death-receptor signaling, have been shown to be required for this antiviral pathway.^72^ FADD deficient or RIP1 deficient MEFs are highly susceptible to VSV infection.^76^ It is demonstrated that TRADD (TNFR-associated death domain) is recruited to ISP-1 to orchestrate complex with FADD and RIP1 to activate NF-κB, as well as TRAF3 and TANK for IRF3 activation.^77^ Silencing expression of TRADD results in impaired RIG-I-mediated antiviral responses.^77^

The NLRs are characterized as the N-terminal CARD or pyrin domain, central NACHT (nucleotide-binding and oligomerization) domain, and C-terminal LRRs domain.^78^ NLR family can be divided into three distinct subfamilies: the NODs such as NOD1 and NOD2, the NLRPs (NOD-, LRR- and pyrin domain-containing proteins, also known as NALPs) including NLRP1-14, and the IPAF subfamily.^79,80^ The pyrin-containing NLRs are mainly involved in inflammasome such as NLRP1, NLRP3, and NLRC4, which will be discussed later.^81^ The CARD-containing NOD1 and NOD2 are cytosolic receptors and recognize bacterial components iE-DAP (γ-D-glutamyl-meso-diaminopimelic acid) and MDP (muramyl dipeptide), respectively.^71,82^ Upon stimulation, NOD1 and NOD2 self-oligomerize and recruit RIP2 (also known as RICK and RIPK2) through homotypic CARD–CARD interactions.^83–85^ cIAP1 and cIAP2 mediate K63-linked polyubiquitination of RIP2,^86^ resulting in the recruitment and activation of TAK1 and IKKs, as well as downstream activation of NF-κB.^85,86^ NOD1 and NOD2 also induce activation of MAPK (mitogen-activated protein kinase) pathway through RIP2.^82^

### Activation of canonical NF-κB in the adaptive immune response

T-cell and B-cell are the major components of the adaptive immune system. Upon activation, T- and B-cells undergo proliferation and differentiation into effector cells that mediate different aspects of immune response, including the secretion of cytokines and CTL (cytotoxicity T-lymphocyte) response of T-cells, as well as antibody production by B-cells. Some of activated lymphocytes then differentiate into long-lasting memory cells for rapid and robust immune response encountering the second challenge. Notably, activated CD4^+^ T cells differentiate into distinct effector subsets with different functions including type 1 T helper (Th1), Th2, Th9, Th17, Tfh and regulatory T (Treg) cell,^87–89^ while Tregs also arise during thymic development dependent on TCR signals activated by self-antigen–MHC complexes.^89,90^ Th1 cells produce proinflammatory cytokines IL-12 and IFNγ to activate macrophage and mediate immune response to intracellular pathogens.^87^ Th2 cells release IL-4, IL-5, and IL-13 and stimulate mast cells, eosinophils and basophils in response to helminths. Th17 cells produce IL-17 and IL-22 to recruit neutrophils and mediate immune response against bacteria and fungi.^89^ Th17 also plays a critical role in maintaining intestinal homeostasis. Treg cells produce immunosuppressive cytokines, such as IL-10 and TGF-β, to inhibit immune responses.^87,90^ Similarly, activated naïve CD8^+^ T cells undergo proliferation and differentiation into a variety of effector and memory cell types including T_eff_ (T effector cells), T_scm_ (T memory stem cells), and T_cm_ (T central memory cells), which are responsible to eliminate tumor cell and virus infected cells.^91–93^

T cell activation requires both TCR and costimulatory signals. Upon TCR activation, LCK, belonging to SFK (SRC family kinase), is the first molecule recruited to the TCR-CD3 complex.^94,95^ LCK phosphorylates ITAMs (immunoreceptor tyrosine-based activation motifs) of CD3δ-, γ-, ε- and ζ-chains,^96,97^ enabling the recruitment of ZAP70 (ζ-chain associated protein kinase of 70 kDa) which is sequentially phosphorylated by LCK.^98,99^ Activated ZAP70 phosphorylates LAT (linker for activation of T cells), a transmembrane adaptor protein, which recruits numerous signaling components for signal transduction through three major signaling pathways: Ca^2+^-dependent pathway, the MAPK and NF-κB pathways.^94,100–102^ TCR activates canonical NF-κB through CBM (CARD11/Bcl10/MALT1) complex, which requires PKC-θ- (protein kinase C-θ) mediated phosphorylation of CARD11 (CARD-containing MAGUK protein 1, also known as CARMA1).^103–105^ In brief, activated LAT recruits another scaffold protein SLP76 (SH2 domain-containing leukocyte protein of 76 kDa).^106^ After phosphorylated by ZAP70, SLP76, together with LAT, recruit PLCγ1 (phospholipase Cγ1), which hydrolyses Ptdlns(4,5)P_2_ (phosphatidylinositol-4,5-bisphosphate) to generate DAG (diacylglycerol) and lns(1,4,5)P_3_ (inositol-1,4,5-trisphosphate).^95,107^ The membrane-associated DAG recruits PKC-θ that in turn phosphorylates CARD11.^108–111^ Phosphorylated CARD11 undergoes a conformational change, leading to the recruitment of Bcl10 (B cell lymphoma 10) and MALT1 (mucosa-associated lymphoid tissue lymphoma translocation protein 1) to form CBM complex.^112,113^ MALT1 has the TRAF6-binding motifs and thus recruits TRAF6, which mediates autoubiquitination and K63 ubiquitination of Bcl10,^114^ followed by the activation of TAK1 and IKK-mediated canonical NF-κB activation (Fig. 1).^7,103^ However, TRAF6 deficiency in T-cells does not cause abnormality of NF-κB activation,^115^ suggesting redundancy of other E3s in this pathway. Indeed, MIB2 is identified as a ubiquitin E3 ligase that interacts with BCL10, overexpressed MIB2 causes autoubiquitination and ubiquitination of NEMO, and promotes the recruitment of TAK1.^116^ MIB2-knockdown HEK293T cells show impaired NF-κB activation in NF-κB luciferase reporter assays.^116^ It remains to be studied whether MIB2 is involved in TCR-mediated activation of NF-κB under in vivo conditions.

BCR signaling plays critical role in multiple aspects of B cell biology. The recognition of antigens by BCR is prerequisite for functionally differentiation into high-affinity antibody producing plasma cells and long-lived memory B-cells after a series of reactions in the germinal center.^117,118^ The BCR is composed of heavy and light chains of membrane Ig (immunoglobin), associated with the Igαβ heterodimer, which contains ITAMs.^119–121^ The molecular mechanism of BCR signaling is highly similar with TCR signaling. Both TCR and BCR active canonical NF-κB through CBM complex, while PKC-β in B-cells, instead of PKC-θ in T cells, is upstream of CBM.^111,122,123^ In B-cells, after receptor engagement, the SFKs including LYN, FYN and BLK (B-lymphoid tyrosine kinase) phosphorylate ITAMs on Igα and Igβ chains, followed by recruitment and activation of SYK (cytosolic tyrosine kinase).^124–126^ SYK then phosphorylates the cytosolic adaptor protein SLP65 (also known as BLNK),^127,128^ which recruits PLCγ2 and mediates downstream signal transduction toward canonical NF-κB, similar to TCR signaling pathway.^124,127,129^

### Activation of canonical NF-κB by proinflammatory cytokines

Upon activation, canonical NF-κB induces the production of proinflammatory cytokines such as TNF-α and IL-1β in the innate immune system, resulting in inflammatory response. On the other hand, these proinflammatory cytokines activate canonical NF-κB. The IL-1 receptor family shares an intracellular TIR domain with TLR, which is involved in the initiation of signaling.^40,130,131^ Thereby IL-1R and TLRs share similar signaling pathways. In brief, upon binding to IL-1, the IL-1RI (the receptor of IL-1) recruits the TIR-domain-containing adaptors MyD88 to the receptor,^132^ followed by the recruitment of IRAKs and TRAF6.^130,133,134^ TRAF6 mediates K63-linked autoubiquitination,^135^ leading to the activation of TAK1 complex.^49,136^ Activated TAK1 phosphorylates and activates IKKs as well as subsequent canonical NF-κB pathway,^137,138^ which induces the expression of proinflammatory cytokines. Meanwhile, TAK1 activates the MAPK signaling, resulting in the activation of transcription factor AP-1, which also induces the expression of cytokines.^40,138^

Upon TNF-α binding to its receptor TNFR1, which is characterized containing a DD (death domain) in cytoplasmic tails, TNFR1 recruits TRADD through homophilic interactions between DD domains, and drives assembly of the E3 ubiquitin ligases cIAP1 and cIAP2 as well as TRAF2 with the protein kinase RIP1.^139–141^ RIP1 is then K63-ubiquitinated and recruited to NEMO, resulting in the formation of TAK1-IKK complex and activation of IKK as well as downstream signals^142,143^ (Fig. 1). In addition to NF-κB, TNFR1 triggers signaling to apoptosis through a complex composed of TRADD, RIPs, FADD and caspase-8.^144,145^ These two signaling pathways have opposite consequences with cell survival or cell death. And in most cell types, TNFR1 induces the activation of NF-κB rather than apoptosis.^145,146^ The activity of apoptosis complex is normally inhibited by c-FLIP, a caspase-8 homolog that competes with caspase-8 to interact with FADD,^147^ while NF-κB drives the expression of c-FLIP and other anti-apoptotic proteins including cIAP1 and cIAP2,^148–151^ thus cell survives.

### Canonical NF-κB and inflammasome

The production of proinflammatory cytokines, such as TNF-α and IL-6, is induced by canonical NF-κB directly, meanwhile, the production of IL-1β is regulated in two steps: transcription and maturation.^39^ IL-1β is first induced as an inactive precursor named pro-IL-1β by canonical NF-κB.^152^ Pro-IL-1β is then cleaved by active caspase-1, a cysteine protease that is involved in the inflammasome complex.^79,153,154^ Inflammasomes are multiple protein complexes assembled upon cellular infection or stress that regulate the maturation of the proinflammatory cytokines such as IL-1β and IL-18, leading to inflammation and immune defense.^79,155^ Additionally, inflammasome activation causes pyroptosis, a rapid inflammatory form of cell death.^156–158^ Canonical inflammasomes consist of the inflammasome sensor receptor, the adaptor protein ASC (apoptosis-associated speck-like protein containing a CARD) and caspase-1.^155,159^ Upon activation, the sensor receptor engages ASC, serving as a scaffold to recruit the inactive zymogen pro-caspase-1.^160,161^ Pro-caspase-1 clustering induces caspase-1 self-cleavage and the formation of the active caspase-1, which further cleaves pro-IL-1β and pro-IL-18, generating active IL-1β and IL-18.^155,162^

Most inflammasome receptors are NLR sensors, such as NLRP1, NLRP3, NLRC4 (NOD-, LRR- and CARD-containing 4, also known as IPAF).^163,164^ Besides NLRs, another pyrin domain-containing protein AIM2 (Absent in Melanoma 2) also plays a role in inflammasome assembly.^159,165^ The NLRP3 inflammasome is currently the most characterized inflammasome.^81^ The basal expression of NLRP3 is not sufficient to activate inflammasome, so under most conditions, before exposure to activation signals, NLRP3 requires priming signals, which involve a post-translational regulatory mechanism and a NF-κB-dependent transcription regulatory mechanism.^155,166,167^ Canonical NF-κB induces the expression of NLRP3 and pro-IL-1β upon microbial components or cytokines stimulation, serving as NLRP3 priming signal.^152,168^ Additionally, the deubiquitination of NLRP3 also serves as an early priming signal, and inhibition of deubiquitination disturbs human NLRP3 activation.^166,169^ After priming, NLRP3 assembles the NLRP3 inflammasome which cleaves pro-IL-1β to generate active cytokine IL-1β in response to stimuli from fungi, bacterial and viral pathogens, as well as DAMPs released by damaged host cells.^164,170^ Dysregulation of inflammasome leads to various autoinflammatory and autoimmune diseases.^155,171^

### Activation of canonical NF-κB by DNA damage

It has been long known that DNA damage also induces activation of canonical NF-κB. The signal transduction of DNA-damage-induced activation of NF-κB is tightly controlled in spatiotemporal manner. DNA damage induces activation of ATM (ataxia telangiectasia mutated) and a series sequential post-translational modification of NEMO in nucleus, including SUMOylation, phosphorylation and ubiquitination.^172,173^ These modifications of NEMO are essential for signal transduction to activate NF-κB.

SUMOylation is a protein post-translational modification mechanism.^174^ SUMO (small ubiquitin-related modifier) is a group of ubiquitin-like proteins, including SUMO1, SUMO2 and SUMO3, that are conjugated to substrates through activating enzyme (SAE1/SAE2), conjugating enzyme (UBE21) and ligase, suggesting a similarity of SUMOylation and ubiquitination on the regulating mechanism and biological function.^175^ Upon genotoxic stress, NEMO is modified by SUMO1 at K277 and K309, which is mediated by PIASy (protein inhibitor of activated STATy).^176,177^ SUMOylation of NEMO prevents its nucleus export. Mutations of K277 and K309 abolish the SUMOylation of NEMO and activation of NF-κB induced by DNA damage.^177^ The mutated NEMO displays largely cytoplasmic localization following DNA damage,^177^ suggesting essential role of SUMOylation for nucleus localization of NEMO.

In parallel, the phosphatidylinositol 3-kinase like protein ATM is activated upon DNA damage. Activated ATM then phosphorylates SUMOylated NEMO at S85 (serine 85),^178^ leading to removal of SUMO1 and ligation of monoubiquitin of NEMO at K277 and K309. Ubiquitinated NEMO, together with ATM, translocate from nucleus to cytoplasm and activate downstream IKKs. Either siRNA knockdown of ATM or S85A mutant of NEMO leads to impaired ubiquitination of NEMO,^177,178^ suggesting that ATM and phosphorylation of NEMO are prerequisite of monoubiquitin of NEMO. In contrast to SUMOylation, ubiquitination of NEMO promotes nucleus export of NEMO, as NEMO-S85A is retained in nucleus upon DNA damage and a ubiquitin fusion to NEMO-S85A restores NEMO cytoplasmic distribution.^178^ In cytoplasm, TAK1 is involved in ATM-NEMO-mediated NF-κB activation in response to DNA damage.^179,180^ The scaffold protein ELKS (a protein rich in glutamate, leucine, lysine, and serine) is K63-linked ubiquitinated by ubiquitin ligase XIAP ((X-linked inhibitor of apoptosis), which is ATM-dependent.^180^ The ubiquitination of ELKS facilitates the formation of TAK1, NEMO and IKK complex,^180^ resulting in activation of canonical NF-κB.

## Regulation of canonical NF-κB

Canonical NF-κB is activated rapidly, inducing numerous proinflammatory mediators and molecules that lead to inflammatory response as well as activation and differentiation of immune cells. However, aberrant activation of NF-κB cause chronic inflammation, oncogenesis and autoimmune disease. Thus, NF-κB is tightly regulated to maintain the homeostasis.

### Negative regulation of canonical NF-κB

The activity of canonical NF-κB is negatively regulated at different levels.^181^ Nuclear localization of NF-κB dimers is required for their transcriptional activity. *Nfkbia* (coding IκBα) and *Nfkbie* (coding IκBε) are NF-κB target genes, serving as a negative feedback regulatory mechanism.^28,29,33^ Newly synthesized IκBα protein binds and drives NF-κB dimer from nucleus back into cytosol, leading to the termination of NF-κB transcriptional activity.^28^ Notably, IκBβ counteracts the inhibitory function of IκBα through binding to RelA and c-Rel in nucleus.^35,182^ Once RelA and c-Rel are bound by hypophosphorylated IκBβ, p50/RelA and p50/c-Rel complex are resistant to IκBα-mediated inhibition, so the transcription of target genes is prolonged.^35,182^ IκBε provides a delayed negative feedback regulation, by a delayed expression compared with IκBα expression.^183^ Notably, IκBε was shown to dampen IκBα-mediated oscillations because of a dual and antiphase working system.

At the transcriptional factor level, NF-κB dimers are also degraded to impair NF-κB activity. IKKα mediates degradation of RelA- and c-REL-containing complex. In LPS-stimulated macrophage, IKKα phosphorylates RelA at Ser536, leading to accelerated turnover of RelA.^184,185^ ECS (elongin-B-elongin-C-cullin-SOCS1) ubiquitin ligase complex facilitates the ubiquitination and subsequent degradation of RelA with the help of COMMD1 (copper metabolism (Murr1) domain containing 1) that promotes the association between SOCS1 (suppressor of cytokine signaling-1), RelA and Culin-2.^186–188^ In addition, E3 ubiquitin ligase PDLIM2 (PDZ and LIM domain protein 2) is also reported to inhibit NF-κB transcriptional activity by removing RelA from DNA binding sites and mediating its degradation.^189,190^ Similarly, following phosphorylation by IKKα, PIAS1 (protein inhibitor of activated STAT 1) moves to the promoter region of NF-κB target genes and inhibits the binding of RelA-containing dimers to DNA at the early phase of NF-κB activation.^191–193^

Deubiquitination plays an important role in the negative regulation of signal transduction upstream IKK. Notably, in all signal pathways to activate NF-κB mentioned above, the signal-induced and reversible K63-linked ubiquitination of scaffold proteins is a prerequisite for canonical NF-κB activation.^26,27^ *Tnfaip3* is a direct NF-κB target gene encoding the DUB (deubiquitinase) A20 that mediates a negative feedback regulation of canonical NF-κB. A20 contains a DUB domain and a C2-C2 zinc finger E3 ubiquitin ligase domain.^9^ The DUB domain of A20 removes the K63-linked ubiquitin chains of RIP1, TRAF6 and NEMO, leading to the disassembly of IKK complex and down-regulation of inflammatory response.^181,194–197^ Additionally, A20 utilizes its additional E3 ubiquitin ligase domain together with another HECT ubiquitin ligase Itch to mediate K48-linked ubiquitination of RIP1 after the removal of K63-ubiquitin chain, resulting in degradation of RIP1 and deactivation of signaling pathways induced by TNF.^198^ Tumor suppressor protein CYLD (Cylindromatosis) is another DUB involved in the negative regulation of canonical NF-κB pathway.^199^ CYLD disassembles K63-ubiquitin chains of several proteins upstream IKKs, including TRAF2, TRAF6, and NEMO, to inhibit activation of IKKs.^199,200^ CYLD-deficient cells show more rapid activation of canonical NF-κB upon TNF stimulation. Taken together, the strength and duration of canonical NF-κB activity are tightly regulated at multiple levels.

### Positive regulation of canonical NF-κB

Canonical NF-κB is also positively regulated, especially by TRAF-mediated polyubiquitination and LUBAC- (linear ubiquitin chain assembly complex) catalyzed linear (M1-linked) ubiquitination.^201^ Ubiquitination is a protein post-translational modification that regulates numerous cellular processes.^103,202^ Ubiquitin, a small protein with 76 amino acids, covalently conjugates to lysine (K) residues of substrate proteins. Ubiquitin conjugation is mediated by a three-step enzymatic process involving first ATP-dependent activation by the E1 (ubiquitin-activating enzyme), subsequent conjugation by E2 (ubiquitin-conjugating enzymes), and finally ligation by E3 (ubiquitin-ligating enzymes).^203–205^ Most of the specificity of ubiquitination is mainly determined by E3s. Human genome encodes over 600 E3s, which serve as catalytic intermediates in ubiquitination (HECT domain E3s) or directly catalyze the transfer of ubiquitin from E2 to substrates (RING or RING-like domain E3s).^206,207^ There are eight different linkage types of ubiquitination, including K6, K11, K27, K29, K33, K48, K63, and M1, in which the C terminus of ubiquitin is attached to lysine residues or the N-terminal methionine (M1) of another ubiquitin.^204,205,208^ Ubiquitin forms monoubiquitination chain and polymeric ubiquitin chain, leading to distinct signaling outcomes for the substrate proteins.^209,210^ It has been well established that K48-linked polymeric ubiquitin chain functions to target substrate proteins for degradation by the 26S proteasome, while M1-and K63-linked ubiquitin chains are involved in signal transduction.^211–213^ Ubiquitination is a fundamental protein post-translational modification mechanism for diverse biological processes including different aspects of immune response.^103,210^

In IL-1R and TLR signaling pathways, TRAF6 acts with E2 enzyme UBC13 and UEV1A to synthesize K63-linked polyubiquitin chain on NEMO and TRAF6 itself.^47,49,137^ The adaptor proteins TAB2/3 bind K63-linked ubiquitin chains through NZF (zinc-finger) domain to activate TAK1.^214^ Mutation of NZF domain abolishes the activation of TAK1 and IKK, while the replacement of NZF domain with a heterologous ubiquitin binding domain recovers the activation of IKK,^215^ indicating the essential role of ubiquitin signals for canonical NF-κB activation. However, the role of TAB2 in activation of canonical NF-κB is argued by the study that macrophages and B-cells lacking TAB2 and TAB3 show little defect of canonical NF-κB activation in response to TLR stimulation,^216^ suggesting the possible redundant adaptor proteins.

In TNFR signaling, The K63-linked ubiquitination of RIP1 at lysine 377 is critical for IKK activation. RIP1 deficient cells fail to activate NF-κB, and reconstitution of RIP1 with K377R mutation is insufficient to rescue IKK activation upon TNF stimulation.^142^ Additionally, the recruitment of TAK1 and IKKs to TNFR is prevented in TNF-stimulated cells with K377R mutation of RIP1. It has been reported that the K63-linked ubiquitination of RIP1 is mediated by cIAP1/2, as the polyubiquitin conjugated to RIP1 is impaired in cIAP1/2 knockdown cells by siRNA upon TNF stimulation.^217^ However, it has also been reported that TNF induced ubiquitination of RIP1 is defective in TRAF2 deficient cells.^218^ Collectively, the ubiquitination plays critical roles in the activation of canonical NF-κB.

Linear ubiquitination mediated by LUBAC is critical for activation of IKKs. Similar with the K63-linked ubiquitination for activation of TAK1, linear ubiquitination chain bound by NEMO is crucial for IKKs activation.^103,219,220^ Linear ubiquitin chains but not K63-linked ubiquitin chains is bound by the UBAN (ubiquitin binding in ABIN and NEMO) motif of NEMO,^143^ leading to its conformational change, which modulates the interaction between NEMO and IKKs, to activate NF-κB.^143^ Although full-length NEMO also binds K63-linked ubiquitin chains, it preferentially binds the linear ubiquitin chains in vitro.^221^ NEMO with mutation in UBAN shows defective interaction with both linear and K63-linked ubiquitin chains, resulting in impaired activation of NF-κB upon TNFR and IL-1RI stimulation, which is associated with human EDA-ID (ectodermal dysplasia with immunodeficiency) diseases.^222^

## Canonical NF-κB in cancer

The function of NF-κB in the immune system has been well studied. The canonical NF-κB pathway participates in almost all immune responses and various diseases. The human diseases caused by mutation of crucial components of NF-κB pathway are summarized in Table 1.

**Table 1:** Roles of NF-κB pathway in human diseases

Protein name	Gene name	Type of disorder	Genetic mutations	Immunological defects in patients	Reference
NEMO	*IKBKG*	Incontinentia pigmenti (IP)	c.184C>T (p.Arg62Ter) c.1219A>G (p.Met407Val) c.1259A>G (p.Ter420Trp)	Failures of NF-κB induction in integument	^399^
			EX4-10DEL	The IP-associated male lethality	^400^
NEMO	*IKBKG*	Hypohidrotic ectodermal dysplasia and immunodeficiency (HED-ID)	c.509T>C c.1167dupT	Reduced memory B cells; failed to differentiate into plasma cells in response to CpG	^401^
			c.1161dupC	Abnormally high levels of IgD and IgE	^402^
			c.1217A>T (p.Asp406Val) c.1249T>C (p.Cys417Arg) c.1250G>T (p.Cys417Phe)	Suppression of IL-2 induction; impaired NF-κB activation; hyper-IgM syndrome; reduced memory B cells	^403–405^
			c.458T>G (p.Leu153Arg) c.1207C>T (p.Gln403Ter)	Impaired NK cell cytotoxic activity	^406^
			c.185G>A (p.Arg62Gln)	N/A	^407^
			c.931G>A (p.Asp311Asn)	N/A	^408,409^
NEMO	*IKBKG*	Osteopetrosis and lymphedema- hypohidrotic ectodermal dysplasia, and immunodeficiency (OL-HED-ID)	c.470A>C (p.Gln157Pro)	Hypogammaglobulinemia (IgG, IgM and IgA) and mixed T- and B-cell dysfunction	^410^
			c.1182-1183delTT	Increased number of CD4 T cells and B cells with normal CD8 T cells; hyper-IgM syndrome	^411^
NEMO	*IKBKG*	Anhidrotic ectodermal dysplasia with immunodeficiency (EDA-ID)	c.863C>G (p.Ala288Gly)	Reduced TNF-α and LPS-induced NF-κB activation	^412^
			c.768+5G>A	Impaired NF-κB activation as IκBα degradation	^413^
			IVS4+866C>T	Impaired NF-κB activation as a generation of frameshift IκBα	^414^
			4.4-KB DUP	Reduced naïve-phenotype T cells and mitogen-induced proliferation of PBMC; increased levels of IgG and IgA; defect in LPS-induced NF-κB response	^415^
			c.1167insC	Deficient cellular immunity	^416^
NEMO	*IKBKG*	Osteopetrosis and lymphedema-anhidrotic ectodermal dysplasia with immunodeficiency (OL-EDA-ID)	c.1259A>G (p.Ter420Trp)	Poor inflammatory reponse; impaired IL-1β, IL-18 and LPS-induced NF-κB activation	^399,417^
			c.1238A>G (p.His413Arg)	Defect in LPS, IL-1β, and TNF-α-induced NF-κB activation; low NK and B memory cell counts	^418^
IκBα	*NFKBIA*	Anhidrotic ectodermal dysplasia With immunodeficiency (EDA-ID)	c.96C>G (p.Ser32Arg) c.95G>A (p.Ser32Asn)	Defective in memory T, B cells and Treg	^419^
			c.107C>A (p.Ser36Tyr)	Reduced TLR/IL-1 and TNFR response to stimuli; reduction in γδ T and effector memory CD8 T cells	^420,421^
			c.110T>G (p.Met37Arg)	Hyper-IgM syndrome with high/normal levels of IgM and low/absent levels of IgA, IgG and IgE; reduced memory B cells	^422^
			c.110T>A (p.Met37Lys)	Decreased number of IL-17 producing T cells; impaired response to LPS and NF-κB activity	^423^
			c.40G>T (p.Glu14Ter)	Defective in production of TNF-α and IL-12 of monocytes and IFN-γ of T cells	^424^
			c.32G>A (p.Trp11Ter)	Impaired cytokine production in response to TLR ligands;	^425^
			c.95G>T (p.Ser32Ile)	impaired response to TLR, IL-1β, IL-18 and TNFR; defective in memory T cells	^426^
			p.Gln9Ter	Impaired response to LPS-induced NF-κB activity	^427^
IKKα	*IKBKA*	Severe fetal encasement malformation	c.1264C>T	Embryonic lethal	^428^
IKKβ	*IKBKB*	Severe combined immunodeficiency (SCID)	c.1292dup	Impaired immune response to stimulation; absent of regulatory T cells and γδ T cells	^429^
			c.814C>T (p.Arg272Ter)	Hyper-IgM syndrome	^430^
			c.607G>A (p.Val203Ile)	Immune dysregulation; deficiency of T and B cell	^431^
P105/50	*NFKB1*	Common variable immune deficiency (CVID)	c.730+4A>G	B cell dysfunction	^432^
			c.491delG (p.G165A)	Hypogammaglobulinemia, decreased frequencies of class-switched B-cells and impaired T-cell proliferation	^433^
			c.1149delT (p.Gly384Glu)	Hypogammaglobulinaemia with reduced B cells; excessive production of proinflammatory cytokines (IL-1β, TNF-α)	^434^
CYLD	*CYLD*	Familial cylindromatosis	c.547C>T	Familial Behcet-like autoinflammatory syndrome	^435^
			c.1392dup (p.Gly465TrpfsX10)	Cell hyperproliferation	^436^
			c.2252del	Cell hyperproliferation	^437^
CARD9	*CARD9*	Chronic mucocutaneous candidiasis	c.883C>T (p.Gln295Ter)	N/A	^438^
CARD9	*CARD9*	Deep dermatophytosis	c.865C>T (p.Gln289Ter)	Low numbers of Th17 cells	^439^
			c.184G>A c.288C>T	N/A	^440^
CARD11	*CARD11*	B-cell expansion with NF-κB and T-cell anergy	c.368G>A (p.Gly123Asp)	Severe polyclonal B lymphocytosis	^441^
			c.146G>A (p.Cys49Tyr)	Splenomegaly and profound polyclonal B-cell lymphocytosis; elevated transitional and mature naive B cells; few circulating class-switched/memory B cells	^442^
			p.Glu127Gly p.Gly116Ser	Hereditary polyclonal B cell lymphocytosis	^443^
			coiled-coil domain mutants	Diffuse large B cell lymphoma (DLBCL)	^444^
CARD11	*CARD11*	Immunodeficiency	deletion of exon 21	Profound combined immunodeficiency	^445^
			c.2833C>T (p.Gln945Ter)	Agammaglobulinemia; deficient T-cell function; normal T and B lymphocytes	^446^
			c.2923C>T (p.Arg975Trp) p.Glu57Asp p.Leu194Pro	Low production of the cytokine IFN-γ	^447^
CARD14	*CARD14*	Psoriasis	c.467T>C (p.Leu156Pro) c.349þ1G>A (p.Glu156del) c.412-414del	N/A	^448^
			c.112C>T (p.Arg38Cys) c.424G>A (p.Glu142Lys) c.425A>G (p.Glu142Gly) c.511C>A (p.His171Asn) c.536G>A (p.Arg179His) c.571G>T (p.Val191Leu) c.599G>A (p.Ser200Asn) c.854A>G (p.Asp285Gly) c.1778T>A (p.Ile593Asn) c.349G>A (p.Gly117Ser) c.413A>C (p.Glu138Ala)	Complex interplay between keratinocytes, skin resident immune cells and infiltrating leukocytes, including neutrophils, macrophages, conventional and plasmacytoid dendritic cells	^449–451^
MALT1	*MALT1*	Combined immunodeficiency (CID)	c.266G>T (p.Ser89Ile)	Normal numbers of T and B lymphocytes; impaired cellular and humoral immunity	^452^
			c.1739G>C (p.Trp580Ser)	Severe dermatitis, severe inflammatory gastrointestinal disease and pneumonia	^453^
BCL10	*BCL10*	Combined immunodeficiency (CID) + autoimmunity	c.57+1G>A	Defects in both hematopoietic and non-hematopoietic immunity	^454^
BCL10	*BCL10*	Germ cell tumor and Non-Hodgkin’s Lymphoma	c.499dup c.172C>G c.427-428dup	MALT B Cell Lymphoma	^455,456^
A20	*TNFAIP3*	Behcet-like autoimmunity	c.547C>T	N/A	^457^
			c.680T>A c.671delT (p.Phe224Ser) c.811C>T c.1809delG (p.Thr604Arg) c.918C>G c.799delG (p.Pro268Leu)	Increased egradation of IκBα and nuclear translocation of the p65 subunit; increased production of NF-κB-mediated proinflammatory cytokines	^458^
RIP1	*RIPK1*	Immunodeficiency	c.4del c.21del c.2064del	Immunodeficiency with lymphopenia	^459^
RIP1	*RIPK1*	Autoinflammatory Syndrome	p.Asp324Asn p.Asp324His p.Asp324Tyr	Severe intermittent lymphadenopathy	^460^
NIK	*MAP3K14*	Combined immunodeficiency (CID)	c.1694C>G (p.Pro565Arg)	B-cell lymphopenia; decreased frequencies of class-switched memory B cells; hypogammaglobulinemia; impaired ICOSL expression	^461^
RelB	*RELB*	Combined immunodeficiency (CID)	c.1191C>A (p.Tyr397Ter)	Arrested B cells development; poor production of immunoglobulins; reduced output of thymus	^462^
p100/52	*NFKB2*	Common variable immune deficiency (CVID)	c.2564delA (p.Lys855Ser) c.2557C>T	Childhood-onset hypogammaglobulinemia; autoimmune features	^463^
			c.2556_2563del c.2594A>G (p.Asp865Gly) c.2600C>T (p.Ala867Val) c.2557C>T (p.Arg853Ter)	Severe B-cell deficiency; immunodeficiencies; hypogammaglobulinemia	^464–466^

The role of canonical NF-κB in cancer is complex, with either positive or negative effects in initiation and progression of cancer.^183,223^ Activation of canonical NF-κB is a reaction of host defense for pathogen elimination,^39,224^ on the other hand, activation of canonical NF-κB promotes proliferation, survival, angiogenesis and invasion of tumor cells, contributing to tumor promotion and progression.^183,225^ Here we mainly discuss the tumor-promoting effect of canonical NF-κB and correlation between inflammation and cancer.

### NF-κB, inflammation and cancer

Activation of canonical NF-κB is often linked to the inflammation response to infection and injury, which is a part of host defense.^139,224^ Well-regulated inflammation response is essential for host homeostasis. Tumorigenic pathogens cause chronic infections and inflammation, leading to malignancy.^223,226^ It has been shown that chronic infections and inflammation contribute to certain cancers. For example, the HBV (human hepatitis B virus) is the major risk factor for HCC (hepatocellular carcinoma).^225^ Chronic *Helicobacter pylori* infection is linked to MALT (mucosa-associated lymphoid tissue) lymphoma and gastric cancer.^227^ However, immune dysregulation also causes chronic inflammation, leading to chronic or systemic inflammatory diseases such as RA (rheumatoid arthritis), IBD (inflammatory bowel disease) and psoriasis.^224^ Among these chronic inflammatory diseases, IBD is tightly correlated with colorectal cancer, while RA and psoriasis do not show significant tumor-promoting effect.^228^ There are other factors also contributing to chronic inflammation-related cancer, such as tobacco smoke, silica particles and obesity.^229–231^

Cancer could be considered as a chronic disease that results from the uncontrolled growth of certain type of cells. With the initiation and progression of cancer, several hallmarks in cell physiology are essential, including self-sufficient in growth, resistance of apoptosis, evasion to growth-inhibitory signals, capability of angiogenesis and tissue invasion.^232^ The tumor-promoting functions of canonical NF-κB result from several mechanisms that regulate different characterization of tumor progression. Firstly, inflammatory microenvironment inside of tumor increases mutation rates mainly in a ROS- (reactive oxygen species) and RNI- (reactive nitrogen intermediates) dependent manner, promoting tumor initiation.^227,233,234^ In inflammatory cells such as macrophages (especially tumor-associated macrophages, TAMs) and neutrophils, activation of canonical NF-κB induces the expression of cytokines such as TNF-α, IL-1β, and IL-6 that promote the proliferation of malignant cells and tumor stroma cells.^235–237^ Canonical NF-κB also promotes angiogenesis through regulating pro-angiogenic genes such as VEGF (vascular endothelial growth factor), MCP-1 (macrophage inflammatory protein-1) and CXCL8 (CXC-chemokine ligand 8, also known as IL-8), thereby facilitating tumor invasion.^238–240^ Additionally, activation of NF-κB and other transcription factors such as STAT3 and AP1 (activator protein 1) induces expression of chemokines that recruit more immune cells, including macrophages, DCs, mast cells, neutrophils and T- and B-cells, further aggravating inflammatory response.^240,241^ Besides, for malignant cells, proinflammatory cytokines such as TNF-α and IL-1β activate canonical NF-κB, leading to expression of anti-apoptotic genes such as BCL-X_L_ (B-cell lymphoma X_L_) and BCL2 (B-cell lymphoma 2), as well as the caspase inhibitor cIAPs, thereby promoting the survival of tumor cells and cancer progression.^236,242–244^

For example, in a CAC (colitis-associated cancer) mouse model, mice with deletion of IKKβ in enterocytes (exposed to AOM and DSS to induce tumor development) show dramatically decreased tumor incidence, but no reduction of tumor size.^245^ IKKβ-deficient enterocytes undergo apoptotic after a few days of exposure to AOM (azoxymethane) and DSS (dextran sulfate sodium salt), probably due to impaired expression of BCL-X_L_.^245^ In addition, the deletion of IKKβ in myeloid cells leads to reduction in tumor number and tumor size, a consequence of impaired proliferation caused by reduced expression of NF-κB-dependent proinflammatory cytokines production in myeloid cells.^245^

### Cancer therapy by targeting NF-κB

Given the tumor-promoting role of canonical NF-κB in cancer, selective inhibition of canonical NF-κB might be applied in clinical therapy. Several anti-inflammatory drugs such as aspirin, sodium salicylate and dexamethasone have been proved to suppress NF-κB activation.^246–248^ Aspirin and sodium salicylate are shown to inhibit NF-κB by blocking degradation of IκBα and thereby inhibiting NF-κB.^249,250^ In addition to inhibition of key components of the canonical NF-κB pathway, an alternative choice is to block its downstream targets or upstream stimulators. For example, TNF-α is the activator and effector of NF-κB pathway. Anti-TNF-α antibody has been applied in phase I and II clinical cancer trials, and evaluated as partial disease stabilization effects.^251–253^ The current anti-TNF-α antibodies approved by FDA include infliximab, adalimumab and golimumab.^254^ Infliximab is proved to be well tolerated without dose-limiting toxic effects in advanced cancer by clinical studies,^255^ and seven in forty-one patients treated with infliximab show disease stabilization.^255^

Blockage of NF-κB alone might be not sufficient for cancer regression. Combination with NF-κB inhibitors and conventional therapies such as chemotherapy and radiotherapy could be more effective. Besides, clinical therapy could take advantage of NF-κB inhibitors and inhibitors of other signaling involved in inflammation such as AP1 and STAT3 to develop effective and specific therapy for certain cancers. It is well established that canonical NF-κB plays essential roles in immune response, thus long-term use of NF-κB inhibitor could result in immunodeficiency, so NF-κB inhibitor for cancer therapy should be used in a short period of time. Additionally, an ideal NF-κB inhibitor should only target NF-κB pathway without effects on other signaling pathways. Nevertheless, it is a long way to go for the application of NF-κB inhibitor in cancer therapy.

## Regulation of non-canonical NF-κB pathway

RelB/p52 heterodimers are considered as the transcription factors of non-canonical NF-κB pathway. p100 is the precursor of p52 that functions as IκB-like protein to preferentially block the translocation of RelB to nucleus. The proteolytic processing of p100 results in the production of p52 and liberation of RelB, forming a RelB/p52 dimer that translocate to nucleus. So, the processing of p100 is the central event of non-canonical NF-κB signal pathway, which is regulated by NIK-IKKα axis.^1–3^

### Phosphorylation, ubiquitination, and processing of p100

**Fig. 2:**
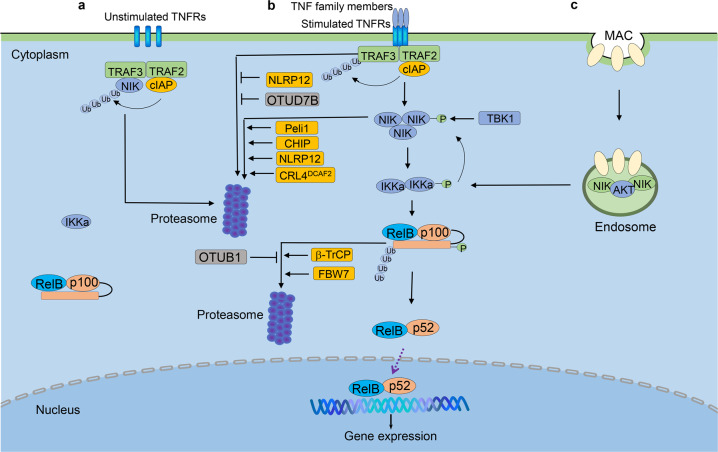
Regulation of non-canonical NF-κB pathway. **a** In steady-state, newly synthesized NIK is targeted for ubiquitination-dependent degradation mediated by the cIAP-TRAF2-TRAF3 E3 ubiquitin ligase complex, which prevents NIK accumulation and non-canonical NF-κB activation. **b** Ligation of specific TNFR superfamily members by their ligands (TNF family members) induces the recruitment of TRAF3-TRAF2-cIAP to the receptor complex, followed by K48 ubiquitination and degradation of TRAF3, resulting in stabilization and accumulation of NIK. NIK, together with IKKα, mediates p100 phosphorylation and ubiquitination-dependent process, to generate p52 and allow p52-RelB heterodimer to enter the nucleus for target gene transcription. **c** Activation of non-canonical NF-κB by MAC involves the formation of an endosome-based signaling complex containing NIK, AKT and MAC. AKT mediates NIK stabilization and IKKα phosphorylation and activates downstream non-canonical NF-κB. Non-canonical NF-κB is negatively regulated through TRAF3 deubiquitination mediated by OTUD7B and degradation of NIK mediated by IKKα, TBK1, CHIP, NLRP12, Peli1 and CRL4^DCAF2^. NIK, NF-κB-inducing kinase; TRAF3, TNFR-associated factor 3; TRAF2, TNFR-associated factor 2; CHIP, carboxyl terminus of HSC70-interacting protein; NLRP12, nucleotide-binding domain and leucine-rich-repeat containing proteins 12; cIAP1/2, cellular inhibitor of apoptosis 1 and 2; MAC, complement membrane activation complex

In the steady-state, p100 is barely converted to p52. The processing of p100 is inhibited by its C-terminal PID (processing-inhibitory domain) and an ARD (ankyrin repeat domain). p100 with C-terminal truncation mutation (p100ΔC) is under a low level of constitutive processing and nuclear translocation.^1,256,257^ PID has a death domain (DD), and the mutation in DD abolishes the inhibitory function of PID. ARD masks the nuclear translocation sequence in the N-terminal RHD and suppresses p100 nuclear translocation with the help of DD.^1,256,257^ The processing of p100 is phosphorylation- and ubiquitination-dependent. The C-terminal of p100 contains a NIK-responsive sequence similar to the phosphorylation site of IκBα. Two serine residues (Ser866 and Ser870) have been identified as the key residues that are phosphorylated by NIK-IKKα and are required for p100 processing (Fig. 2).^1,15^ The domain containing the p100 phosphorylation site is similar to the consensus-binding domain of β-TrCP, the ubiquitin E3 ligase that is also involved in the ubiquitination-dependent degradation of phosphorylated-IκBα.^258^ Studies using genetic and synthetic chemistry methods reveal that the phosphorylated Ser866 and Ser870 contain a β-TrCP binding site, and SCF^β-TrCP^ serves as the ubiquitin E3 ligase to mediate inducible p100 processing (Fig. 2).^259^ It is worthy of mentioning that the constitutive processing of p100ΔC is SCF^β-TrCP^ independent since p100ΔC mutants do not bind β-TrCP.^260^

Like p105, p100 is processed in the basal and inducible manner.^1,3^ SCF^β-TrCP^ is the major ubiquitin E3 ligase that induces p100 processing in a NIK-dependent manner. TRIM9, a ubiquitin E3 ligase specifically expressed in brain tissue, inhibits NIK induced p100 processing, which is also β-TrCP dependent. Since TRIM9 also attenuates canonical NF- κB activation by competing with IκBα to bind to β-TrCP, it is likely that TRIM9 also interferes with the interaction between p100 and β-TrCP.^261,262^ Another E3 ligase, Fbw7, regulates p100 degradation by mediating p100 ubiquitination dependent on GSK3β-mediated phosphorylation.^263^ The location of p100 is crucial for Fbw7-induced degradation since Fbw7 preferentially reduces p100 level in the nucleus.^264^ As mentioned above, β-TrCP binds to phosphorylated p100 (Ser866 and Ser870), as well as the phosphorylated IκBα and p105 upon stimulation. However, Fbw7 specifically interacts with p100, but not p105 or IκBα, and phosphorylation at Ser707 and Ser711 of p100 is required for interaction of Fbw7 and p100.^263–265^ Otub1, a deubiquitinase, inhibits K48-linked ubiquitination of p100, that results in stabilization of p100 in both signal-induced and steady states^266^ (Fig. 2). Together, it is a reasonable hypothesis that the fate of p100, degradation or processing, may be determined by phosphorylation sites targeted by kinases activated by different stimuli, as well as by the colocalization with specific ubiquitin E3 ligases. A certain proportion of p100 is also conjugated with SUMO1 mediated by SUMO-conjugating enzyme Ubc9, which is essential for p100 process.^267^ It is interesting to determine whether there is any common mechanism shared by ubiquitination and SUMOylation to regulate p100 process.

### NIK and IKKα as the critical kinases in non-canonical NF-κB pathway

NIK, also known as MAP3K14, is the first identified component of the non-canonical NF-κB pathway; indeed, it has been initially implicated in NF-κB activation upon TNF receptor pathway.^268,269^ Further research indicates that *Map3k14* deficiency or mutation of alymphophasia (*Aly*) has little effect on TNF-α-induced NF-κB activation but completely blocks the p100 processing.^270^ Overexpression of NIK induces phosphorylation, sequential ubiquitination and processing of p100^15^. So far, all the identified non-canonical NF-κB inducers are capable of activating NIK, establishing NIK as the central node of non-canonical NF-κB pathway.^12^ While NIK induces phosphorylation of p100, it does not directly phosphorylate p100 in vitro. Instead NIK mediates phosphorylation and activation of IKKα. Transgenic mice with inactivated IKKα share similar but not identical phenotypes with NIK KO mice or *Aly/Aly* mice,^16,271^ suggesting the crucial role of IKKα in the non-canonical NF-κB pathway. Although it is well accepted that p100 is the direct substrate of IKKα, IKKα does not induce p100 processing as effectively as NIK. One reason might be that the binding of IKKα and p100 requires NIK. Other mechanism(s) beside NIK could also be important to orchestrate the non-canonical NF-κB pathway. For example, phosphorylation of IKKα at Thr23 by MAP3K8 (also known as COT, Cancer Osaka Thyroid) is also indispensable for p100 processing and RANKL-induced osteoclastogenesis, which is further promoted by AKT.^272,273^ The precise mechanism by which IKKα regulates non-canonical NF-κB signaling remains to be fully understood.

### TRAF-dependent and TRAF-independent degradation of NIK

NIK was initially found to bind TRAF2 upon activation of canonical NF-κB induced by TNF receptor and IL-1 receptor.^268^ Subsequent yeast two-hybrid screening finds that TRAF3 strongly binds NIK through the N-terminal region. Newly synthesized NIK is bound by TRAF3 for ubiquitination-dependent degradation, which keeps NIK protein at the extremely low level in the steady-state (Fig. 2). Proteasome inhibition by MG132 treatment results in the accumulation of NIK, indicating TRAF3-mediated degradation of NIK is dependent on ubiquitination-proteasome system.^13^ In transfected cells, TRAF3 inhibits the function of NIK in inducing p100 processing, while knockdown of TRAF3 leads to NIK accumulation and p100 processing.^274^ Activation of non-canonical NF-κB by CD40 and BAFFR involves TRAF3 degradation and NIK accumulation, suggesting a crucial step in the activation of non-canonical NF-κB pathway is to rescue NIK from TRAF3-mediated degradation. The domain of NIK responsible for NIK-TRAF3 interaction is identified as a seven-amino-acid motif (78-84).^13^ The NIK mutant lacking the TRAF3-binding domain, NIKΔ78-84 or NIKΔT3, is stable because the mutant NIK escapes from TRAF3-mediated degradation. The transgenic mice bearing NIKΔT3 mutant develop B-cell hyperplasia, severe autoimmune disorders and tumors in multiple organs.^275^ Although TRAF3 is identified as the negative regulator of NIK, TRAF3 does not catalyze ubiquitination of NIK in vitro, suggesting TRAF3 may function as the adaptor protein rather than direct ubiquitin E3 ligase.^276^ Study has identified cIAP1 and cIAP2 as E3 ubiquitin ligases that mediate NIK ubiquitination.^276^ Indeed, TRAF2/3 and cIAP1/2 form a complex, in which TRAF2 serves as a linker to connect TRAF3 to cIAP1/2 since TRAF3 does not bind cIAPs directly. In this regard, cIAP1/2 are the functional ubiquitin E3 ligases that regulate ubiquitination and degradation of NIK (Fig. 2).^276,277^ TRAF-cIAP-dependent degradation of NIK is the pivotal mechanism to silence non-canonical NF-κB activation in the steady-state. Similar to the function of IKKβ in canonical NF-κB pathway, IKKα is responsible for phosphorylation and induced processing of p100, upon NIK activating signals. However, IKKα also mediates a negative feedback regulation of NIK.^278^ IKKα phosphorylates the C terminus of NIK and causes NIK degradation that is independent of TRAF-cIAP E3 complex, thus serving as a mechanism to prevent the uncontrolled accumulation of NIK and constitutive activation of non-canonical NF-κB pathway.^278^

CHIP (carboxyl terminus of HSC70-interacting protein), with a U-box domain in the C-terminal having ubiquitin E3 ligase activity, binds to NIK via its TPR domain in N-terminal.^279^ In both HEK293 and primary hepatocytes, overexpression of CHIP significantly increases NIK ubiquitination, leading to NIK degradation as well as reduced non-canonical NF-κB activity (Fig. 2). The interaction of CHIP and NIK, but not the ubiquitin E3 ligase activity, is required for NIK ubiquitination, suggesting CHIP might function as an adaptor protein that coordinates with TRAF3.^279^ Whether CHIP is also involved in cIAP-mediated ubiquitination and degradation of NIK, and the function of CHIP in the stimuli-induced activation of non-canonical NF-κB remain unclear.

Another two E3 ligases, CRL4^DCAF2^ and Peli1, are reported to mediate the polyubiquitination and subsequent degradation of NIK to inhibit non-canonical NF-κB activation^280,281^ (Fig. 2). CRL4^DCAF2^ is a critical E3 ligase in cell cycle and mitotic and S phase progression.^282,283^ CRL4^DCAF2^ promotes NIK degradation independent of TRAF-cIAP, which further inhibits IL-23 production in dendritic cells and suppresses the development of psoriasis.^280^ Peli1 functions in many signaling pathways in innate immune cells and T-cells.^284–286^ Like CRL4^DCAF2^, Peli1 interacts with NIK and mediates K48-linked ubiquitination and degradation of NIK. Overexpression of Peli1 inhibits non-canonical NF-κB activation, which could alleviate lupus-like disease.^281^

### TBK1, the positive and negative regulator of non-canonical NF-κB pathway

The function of TBK1 in the anti-virus immune response and IFN-I production has been well studied.^287^ TBK1, together with its homologous kinase IKKε, are IKK-like kinases, suggesting its pivotal function in NF-κB pathway. TBK1 is initially thought to be a TANK associated kinase that is responsible for activation of NF-κB mediated by TNF-α, IL-1 and CD40.^288^ Indeed, TBK1 also functions as a negative regulator of non-canonical NF-κB pathway.^289^ TBK1 deficiency in B-cell enhances immunoglobulin class switch and fecal IgA level. Consistent with the crucial role of the CD40-and TACI-induced non-canonical NF-κB activation in immunoglobulin class switch, TBK1 KO B-cells have enhanced non-canonical NF-κB activation upon CD40, BAFF, and APRIL stimulation, but not LPS stimulation. TBK1 deficiency does not alter TRAF3 degradation upon LIGHT and BAFF stimulation, but causes the elevated NIK protein level in a phosphorylation-dependent way (Fig. 2). Like IKKα, the phosphorylation of NIK by TBK1 also destabilizes NIK, in the TRAF3-independent manner, since NIK152-C and NIK238-C that lack TRAF3-binding site are also degraded by TBK1. However, the 100 amino acid residues in the carboxyl-terminal of NIK are critical and the deletion of this domain leads to resistance to TBK1-mediated degradation of NIK. Ser862, located in the degradation-determination domain of NIK, is identified as the phosphorylation site targeted by TBK1.^289,290^ These data suggest that TBK1 suppresses IgA production through regulating NIK stability, and whether TBK1-induced NIK degradation is dependent on cIAP has to be elucidated.

### NLRP12 regulates TRAF3 and NIK

NLRPs are essential for inflammasome formation, canonical NF-κB and MAPKs activation.^291^ Studies have suggested the regulatory function of NLRP12 (also known as RNO, PYPAF7 and Monarch-1) in canonical and non-canonical NF-κB pathway.^292,293^ Based on the studies using NLRP12-overexpressing THP-1 cell line, NLRP12 is found to suppress CD40-stimulated NIK accumulation through a proteasome-dependent way, and consequent proinflammatory cytokine production. Mechanism studies indicate NLRP12 interacts with both TRAF3 and NIK, affecting their stability (Fig. 2).^294^ NLRP12 deficiency results in hyperactivation of non-canonical NF-κB and the overexpression of chemokines CXCL12 and CXCL13, which are crucial for colonic inflammation and tumorigenesis in the DSS-induced colitis and colorectal cancer (CRC) model. Bone-marrow transfer experiments indicates that the deficiency of NLRP12 in both hematopoietic and non-hematopoietic cells contributes to CRC tumorigenesis, indicating that NLRP12 regulates CRC tumorigenesis in the extrinsic (through inflammation and recruitment of macrophage) and intrinsic (by activating Jun, Akt and other cancer-related pathways) manners.^294^ NLRP12 has ATP hydrolase activity and prevents IRAK1 phosphorylation upon TLR/IL-1R stimulation, attenuating downstream MAPKs and canonical NF-κB activity.^295,296^ Together with data from Kanneganti’s group,^297^ these findings strongly suggest that dysregulated MAPKs, and canonical and non-canonical NF-κB pathways in NLRP12 KO mice are responsible for the CRC tumorigenesis. However, it is unclear how NLRP12 regulates non-canonical NF-κB pathway.

### OTUD7B as the negative regulator of non-canonical NF-κB

Ubiquitination is one major post-translational modification mechanism which regulates both canonical and non-canonical NF-κB pathway at different levels.^103^ The ubiquitination is a reversible process, regulated by ubiquitin E1/E2/E3 enzyme complex and DUBs. There are about 100 DUBs encoded in the human genome, composed of five families, USP, UCH, Joesphin, JAMM/MPN^+^ and OTU family.^298^ Among the OTU family, A20 is well-known for its crucial regulatory function in NF-κB pathway.^9,299^ OTUD7B is an A20-like protein, as initially identified as a NF-κB inhibitor (Cezanne, Cellular zinc finger anti-NF-κB protein), that targets RIP1 downstream of TNFR.^300^ The crystal studies indicate OTUD7B is the first DUB that specifically cleaves the K11-ubiquitin chain.^301,302^ Since the above studies used the overexpression systems, the function of OTUD7B in the physiological condition remained unsolved until the OTUD7B deficient mice are generated. We identified OTUD7B as a DUB that targets ubiquitinated TRAF3 upon stimulation and thus served as a pivotal regulator of the non-canonical NF-κB pathway (Fig. 2).^303^ In contrast with A20-deficient mice, which suffer from spontaneous and uncontrollable inflammation, OTUD7B knockout mice show little defect in survival and development, suggesting the fundamental differences in signaling functions of OTUD7B and A20. OTUD7B deficiency has no appreciable effect on canonical NF-κB activation upon various stimuli. OTUD7B deficiency however causes enhanced non-canonical NF-κB activation in MEFs and B-cells, following treatment with anti-LTβR and BAFF, respectively, which is different from NPLR12 in this regard. Non-canonical NF-κB activators induce the interaction of OTUD7B and TRAF3, thereby down-regulating TRAF3 ubiquitination and degradation and preventing aberrant non-canonical NF-κB activation. Consequently, the OTUD7B deficiency results in B-cell hyper-responsiveness to antigens, lymphoid follicular hyperplasia in the intestinal mucosa, and elevated intestinal immune response against the intestinal bacterial pathogens. These findings establish OTUD7B as a crucial regulator of signal-induced non-canonical NF-κB activation and indicate a mechanism of immune regulation that involves OTUD7B-mediated deubiquitination and stabilization of TRAF3.^303^

### Endosome-dependent activation of non-canonical NF-κB pathway

MAC (complement membrane attack complex) induced by human PRA (panel reactive antibody) in human endothelial cells stabilizes NIK and activates downstream non-canonical NF-κB pathway (Fig. 2).^304^ Compared with non-canonical NF-κB activation stimulated by LIGHT, the ligand of LTβR, MAC leads to NIK accumulation rapidly (within 30 min), suggesting a different mechanism is involved. Knocking-down TRAF3 or cIAP2 does not affect the MAC-induced NIK stabilization; consistently, TRAF3 degradation is not observed. Genome-wide siRNA screening has identified that the endocytosis and vesicular trafficking-related genes are required for MAC-induced NIK stabilization. Proteins involved in the internalization of MAC, like clathrin, AP2, dynamin and Rab5^+^, are required for the recruitment of AKT to the MAC^+^ endosome, which is responsible for NIK stabilization and IKKα phosphorylation. The active form of Rab5 is crucial for recruitment but not for the activation of AKT.^305^ The formation of MAC^+^ endosome, but not TRAFs-cIAPs is crucial to MAC-induced non-canonical NF-κB activation.

## The function of non-canonical NF-κB in the immune system

The non-canonical NF-κB pathway is only activated by limited stimuli, suggestive of its specific function in immune system and immune response.

### The function of non-canonical NF-κB in B-cell

Non-canonical NF-κB regulates B-cell development and function, in both B-cell intrinsic and extrinsic ways.^3,306^ Distinct T-cell zones and B-cell follicles are formed in lymphoid organs through different types of stromal cells that govern the process of immune cell trafficking, activation, localization and antigen access.^307^ Firstly, non-canonical NF-κB is crucial for function and maturation of stromal cell and the development of the lymphoid organ. The stromal cells of peritoneal cavity in *Aly/Aly* and NIK KO mice have reduced VCAM-1 and ICAM-1 expression, resulting in the defective emigration of B-cell (especially B-1 cell) from the peritoneal cavity and accumulation of B-cell in the cavity.^308,309^ Besides, NIK KO mice and *Aly/Aly* mice suffer from lack of LNs (lymph nodes) and PPs (payer’s patches), and abnormal spleen structure.^23,308,310–312^ Homing chemokines CXCL13 (BLC) and CCL21 (SLC) expressed by stromal cells are critical for B-cells and DCs to entry follicles. However, the induction of these two homing chemokines is strongly reduced in RelB deficient mice, resulting in defective splenic microarchitecture.^313^ Whether these defects in lymphoid organ development and structure caused by impaired non-canonical NF-κB signals are B-cell extrinsic is unknown, since only germline knockout mice were used. Indeed, B-cell specific TRAF3 KO mice have severely expanded B-cell compartments with splenomegaly and lymphadenopathy, due to abnormal basal level of non-canonical NF-κB activity, suggesting a B-cell intrinsic function in lymphoid organ development.^314–317^ Additionally, non-canonical NF-κB directly affects B-cell survival. The TRAF3 deficient B-cell survives much longer, but its proliferation is normal, which might be due to the fact that TRAF3 deficiency protects B cells from apoptosis.^316^

GCs (Germinal centers) are structure in lymphoid organ emerging in B-cell follicles where B-cells undergo clonal expansion, SHM (somatic hypermutation), CSR (class switching recombination) and affinity maturation, and differentiate into long-lived plasma cells and memory B-cells.^318–320^ The initiation and maintenance of GC require collaboration of different cells, including B-cells, Tfhs (follicular helper T cells), Tfrs (follicular regulatory T cells), macrophages, FDCs (follicular dendritic cells), and other stromal cells.^307,318^ During the initiation of GC, FDCs and stromal cells provide the GC localization signals, such as CXCL13, to B-cell. Additionally, FDCs secrete BAFF, which activates non-canonical NF-κB in B-cells for survival and proliferation required for GC development and maintenance. One of the critical events during GC formation is the engagement of activated T- and B-cells on the T-B interface, which provides additional activation signals for B- and T-cells, enabling B-cells to proliferate and develop into GC-B cells, also enabling T-cells to differentiate into Tfhs.^321,322^ During this process, ligation of CD40 (expressed by B-cells) and CD40L (expressed by T-cells) is critical, which activates the non-canonical NF-κB pathway.^319,323^ Indeed, GC B-cell specific deletion of both NF-κB2 and RelB leads to the collapsed formation of GCs, which is associated with the impaired cell-cycle entry and T-B cell interaction.^313,324,325^ Signals through CD40 and BAFFR stabilize NIK and activate the non-canonical NF-κB pathway. Indeed, NIK-deficient mice show a significant decrease of germinal center B-cells.^311^ Consistently, *Aly/Aly* mice have impaired GC formation during the antigen-specific immune response, due to the defective function and development of FDC.^308^ On the other hand, mice with B-cell specific deficiency of TRAF3 show spontaneous germinal center B-cells formation.^314–317^ However, cIAP1^-/-^cIAP2^-/-^ B-cells are incapable of forming GC, suggesting the different roles of TRAF3 and cIAP1/2 in GC formation.^326^

Non-canonical NF-κB regulates GC reactions in multiple layers, such as somatic hypermutation, class switching and affinity maturation. After interacting with T-cells at T-B border, activated B-cells proliferate robustly with the acquirement of high rates of mutations in the variable region of immunoglobulin genes driven by AID (activation-induced cytidine deaminase).^327^ The mutated BCRs show different antigen affinities, then engaged by antigens retained on FDC in LZ (light zone) of GC.^321,328^ During affinity maturation, B-cells travel iteratively between the functionally defined DZ (dark zone) and LZ directed by CXCL12 expressed by DZ stromal cells and CXCL13 expressed by DZ stromal cells,^321,329^ resulting in proliferation of the B-cells with high-affinity BCRs and clearing of B-cells with low-affinity BCRs by macrophages. These high-affinity B-cells differentiate to long-lived antibody-producing plasma cells and memory B-cells, launching humoral immune responses.^320,329^

Antibody diversification is essential for the immune response to different types of antigens. Similar to SHM, primary IgD- and IgM-expressing B-cells undergo class switching with the help of AID to generate B-cells with IgG, IgA, IgE isotypes that have different effector functions.^330,331^ Cytokines play a central role in class switching, for example, IL-4 induces IgG1 and IgE while IFN-γ is responsible for IgG3 and IgG2a production,^332,333^ and TGF-β is involved in IgA class switching.^334,335^ TFG-β, together with CD40L is essential for induction of T-cell-dependent IgA class switch,^336–338^ while BAFF and APRIL released by DCs, or LPS along with TGF-β are sufficient for the T-cell-independent IgA class switching.^330,337,339^ The engagement of CD40L, BAFF, APRIL and their ligand, respectively, induce activation of non-canonical NF-κB in B-cells, indicating a critical role of non-canonical NF-κB in IgA class switch, which has been demonstrated in several mouse models. NIK-deficient mice have impaired somatic hypermutation response of B-cells during the antigen-specific immune response and reduced homeostatic IgA production,^308,340,341^ as well as impaired antigen-specific antibody production.^310,311,342^ Consistently, a biallelic mutation of NIK is identified in patients with B-cell lymphopenia, who suffer decreased frequencies of class-switched memory B-cells and hypogammaglobulinemia.^343^ Deletion of NF-κB2 in GC B-cells also causes a dramatic reduction of antigen-specific antibody-secreting cells.^319,344^ Hyper-immunoglobulinemia and enhanced T cell-independent antibody responses are found in B-cell specific TRAF3 KO mice.^316^ Additionally, OTUD7B has been identified as the negative regulator of stimuli-induced, but not the basal level of non-canonical NF-κB activity. OTUD7B deficient B-cells are hyper-proliferative upon BAFF or agonist anti-CD40 stimulation, but not in the LPS or BCR stimulation groups.^303^ The aged OTUD7B KO mice show enhanced lymphoid organ development spontaneously in the colon with greater B-cell follicles. However, the deficiency of OTUD7B is universal, the elevated non-canonical NF-κB activation in the stromal cell could also be responsible since the blockage of LTβR signaling rescues this phenotype.

### The function of non-canonical NF-κB in T-cell

T-cells are vital effectors in the adaptive immune response to eliminate pathogens and are also involved in autoimmune diseases. Activation of T-cells through ligation of TCRs by peptide-MHC molecules presented by APCs (antigen-presenting cells) requires canonical NF-κB but not non-canonical NF-κB pathway.^3,94,107^ However, non-canonical NF-κB mainly functions in T-cell development in the thymus, as well as generation and maintenance of effector and memory T-cells in the periphery.

The thymus is the primary lymphoid organ responsible for the generation of immunocompetent and self-tolerant T-cells with diverse repertoire,^345,346^ which is tightly regulated within a highly organized thymus structure. The thymus structure is mainly supported by TEC, which is consisted of two major subsets, cTECs (cortical TECs) and mTECs (medullary TECs).^345,347^ The maturation of mTEC, especially Aire (autoimmune regulator) expressing mTEC, is essential to present self-antigen to developing T-cells, in order to eliminate self-responsive T-cells.^17,347–349^ Studies using *CD4-cre*/*Mapk3k14*^*F/F*^ mice support that regulation of T-cell development by non-canonical NF-κB pathway in thymus is mainly through a T-cell-extrinsic manner.^350^ T-cell specific NIK deficiency has little effect on thymocyte development. Indeed, lines of evidence have revealed the critical role of the non-canonical NF-κB pathway in TEC maturation and central-tolerance.^351,352^ Deficiency of non-canonical NF-κB inducers (RANK, LTα, LTβ, LIGHT, CD40, etc.), their receptors, and critical components of this pathway results in severe defects of mTEC development, leading to disturbed self-tolerance.^17,347,352,353^ Signals through CD40 and RANK cooperatively regulate the development of mTECs, most notably the Aire^+^ subset, in a TRAF6-NIK-dependent way, which induces both canonical and non-canonical NF-κB.^18^ By using RANK-deficient mice, researchers find that RANK is essential for the development of Aire^+^ mTECs in the embryonic thymus, and partially contributes to the development of mTECs in postnatal thymus. In contrast, deficiency of RANK and CD40 impairs the postnatal development of Aire^+^ mTEC and medullary architecture as well as self-tolerance in a much quicker manner than deficiency of either RANK or CD40 alone, suggesting the synergic functions of RANK and CD40 signals in TEC development and function.^18,354,355^ Additionally, LTβR signaling regulates the function of mTECs by different mechanisms. LTβR deficient mice show normal frequency of Aire^+^ mTEC but the disorganized medullary region and impaired communication between developing thymocytes and mTECs, leading to the defected T-cell selection and autoimmunity.^356^ CCL21-expressing mTECs are responsible for the migration of positively selected thymocytes from cortex to medulla for negative selection. Further studies demonstrate that compromised interaction between developing thymocytes and mTECs might be due to the impaired CCL21-expressing in mTECs in LTβR deficient mice.^347,357^

Mature T-cells, most of which are naïve T-cells, emigrate from thymus to the periphery where they are challenged by pathogens. Once naïve T-cells are activated by antigens presented on APC, they differentiate into effector and memory T-cells. Activated CD4^+^ T cells differentiate into distinct effector subsets including Th1, Th2, Th9, Th17, Tfh, and Treg cell,^87–89^ To date, little literature indicates the function of non-canonical NF-κB pathway in Th1 and Th2 cells. Whether the non-canonical NF-κB pathway regulates the development of Treg is controversial. *Aly/Aly* mice have fewer CD4^+^CD25^+^Foxp3^+^Treg, coincident with the similar phenotype found in NIK KO mice, although the suppressive function of NIK KO Treg is comparable to WT cells.^358^ Further study reveals that NIK KO mice have reduced CD62L^high^CD4^+^CD25^+^ T-cell and increased CD62^low^CD4^+^CD25^+^ T-cell, which causes the impaired suppressive function of Tregs, and autoimmunity.^359^ Work by Li etc. argues that NIK has a T-cell intrinsic role in maintaining the peripheral Treg homeostasis but not in the thymic development of Foxp3^+^ regulatory T-cells.^350^ To investigate whether the function of non-canonical NF-κB in Treg is T-cell intrinsic, single (transferring WT or NIK KO bone marrow into recipients) and mixed bone-marrow (transferring CD45.2^+^ WT or NIK KO with B6.CD45.1/CD45.2 into B6 recipients) chimeric mice were generated. In the WT recipients of NIK KO bone marrow, Tregs development in thymus are comparable with WT controls; however, the peripheral Tregs are decreased, as also seen in the intact NIK KO mice. This decrease in peripheral Treg is T-cell-intrinsic.^358^

Non-canonical NF-κB pathway is necessary for Th17 differentiation, indicated by the reduced EAE symptom in NIK KO mice. Further study using of T-cell specific NIK KO mice shows that this phonotype is T-cell intrinsic. NIK-deficient naïve CD4^+^ T-cells are defective in Th17 differentiation in vitro.^350^ To examine this phenotype in vivo, EAE was induced in WT and T-cell specific NIK KO (NIK-TKO) mice. The NIK-TKO mice were more resistant to EAE. Compared with WT mice, NIK-TKO mice had reduced Th17 proportion in CNS, as well as the IFN-γ producing Th1 cells.^350,360^ One potential mechanism is that NIK regulates the activation of STAT3 in TCR and IL-6R signaling pathways, both of which are crucial for Th17 differentiation. It was also reported that HVEM (herpesvirus entry mediator), a member of TNFRSF and receptor for LIGHT that activates non-canonical NF-κB pathway in NIK-dependent manner, is involved in Th17 differentiation.^361^

The non-canonical NF-κB pathway has also been shown to regulate memory T-cell generation and/or maintenance in a T-cell intrinsic manner, as indicated by a chimeras transplantation experiment using the bone marrow from NIK KO and WT donor mice.^362^ In this study, the T-cell phenotype is examined on day 8 and day 65 after the first LCMV challenge, and five days after the second challenge. Starting from day 8, both NIK KO CD4^+^ and CD8^+^ T cells had reduced CD44^hi^ portion, compared to WT cells. Sixty-five days after LCMV infection, NIK KO T-cells showed significantly impaired LCMV-specific memory T-cell response, and less surviving NIK KO T-cells responding to viral re-challenge, suggesting NIK and non-canonical NF-κB are required to generate and/or maintain memory T-cells.^362^ Further study using *CD4-cre/Map3k14*^*F/F*^ mice has also confirmed that the function of NIK on memory T-cells is T-cell intrinsic.^350^ NIK seems to be dispensable for naïve T-cell activation in vitro as suggested by comparable proliferation and first-run of cytokine production of WT and NIK KO T-cell stimulated by TCR and co-stimulator molecules.^350^ However, NIK does regulate the antigen-stimulated T-cell response in vivo and the antigen-specific T-cell recall response. One possible mechanism involved could be that some costimulatory molecules belonging to TNFRSF (4-1BB, CD137 and OX40 etc.) are up-regulated by activation of T-cells, which are essential for non-canonical NF-κB activation as a second wave of activating signal for generation of effector/memory-like T-cells in vivo. This notion is further supported by a study that revealed that 4-1BB CAR-T shows an enhanced central memory T-cell phenotype with persistent effector function; meanwhile, CD28 CAR-T preferably develops into effector memory cells after transfusion.^363^ Distinct metabolism pathways activated in two types of CAR-T cells might be the underlying mechanism.

### The function of non-canonical NF-κB in DC

DCs serve as antigen-presenting cells to link innate and adaptive immunity by stimulating naïve T-cells after antigen presentation.^364^ LTβR- and CD40-induced signals are pivotal for full activation of DCs and optimization of the immune response, although the defective non-canonical NF-κB activation does not alter the expression pattern of cell surface markers, such as MHC I and MHC II, CD80/CD86, CD40, CD70, and 4-1BBL, etc. NIK deletion in CD11c^+^ cells leads to defective cross-priming of naïve CD8^+^ T-cells without affecting the MHC-II presentation pathway.^365^ Further in vitro study suggests that IL-12p40 secretion is reduced in NIK-deficient DCs treated with α-CD40 antibody, which is critical for efficient antigen cross-presentation.^365^ Overexpression of NIK in DC enhances antigen presentation, Th1 immune response and antigen-specific CTL response in vivo, suggesting overexpressed NIK in DC could be an effective vaccine adjuvant. Additionally, overexpressed NIK also results in enhanced expression of chemokines and cytokines (TNF-α, IL-6, IL-12, IL-15 and IL-8, MIP-1α/β, CCL3, MCP-1, etc.), to initiate the immune response and recruit immune cells.

In addition to antigen presentation, DCs also play an important role in immune tolerance.^366^ *Aly/Aly* mice show impaired development and function of thymic DCs, as well as expression of their costimulatory molecules, in line with reduced CD80^high^ mTECs. The impaired cross-talk between mTEC and DCs, as well as thymocytes, leads to defective self-tolerance.^367^ Ligation of CD40 on DCs induces expression of indoleamine 2,3-dioxygenase (IDO) that promotes self-tolerance by suppressing T-cell proliferation and inducing apoptosis of activated T-cells. Consistently, siRNA specifically knockdown of NIK and IKKα in DCs results in significantly reduced IDO expression in vitro, suggesting CD40-NIK-IKKα singling axis is required for DCs to induce expression of IDO and immune tolerance.^368^

### The function of non-canonical NF-κB in anti-virus infection

Non-canonical NF-κB can also be activated by some viruses. Viral proteins converge upon NIK to regulate NF-κB activity, such as LMP1 (latent membrane protein 1) of Epstein–Barr virus and Tio protein of Herpesvirus ateles. LMP-1 induces p100 processing, while the dominant-negative NIK (aa-624-947) inhibits NF-κB activation induced by LMP-1, suggesting that LMP1 utilizes NIK to induce p100 process and downstream NF-κB activation.^369^ Additionally, Tio prevents NIK from constitutive turnover in Jurkat T-cells and consequently mediates non-canonical NF-κB activation.^370^

It has been examined that the key signal molecules of non-canonical NF-κB pathway, such as TRAF2, TRAF3, NIK and IKKα, are involved in the anti-virus immune response and type I interferon production.^3,371,372^ Phosphorylation and activation of IKKα by NIK are essential for IKKα-mediated IRF3/7 activation. In pDC, the major resource of IFN-I, IKKα interacts with IRF7, to promote the IFN-I production.^371^ IKKα also regulates IFN-I production in cDC (conventional DC) upon TLR7/9 stimulation by similar mechanism.^373^ However, it remains to be studied whether NIK-IKKα activated non-canonical NF-κB plays a role in IFN-I production.^374^

In the VSV-infection model, NIK KO mice are more resistant to viral infection, which is due to elevated IFN-I production but not IFNAR (type I IFN receptor) signal. The mechanism study indicates that non-canonical NF-κB members NIK and p52 suppress IFN-I production epigenetically. Activation of non-canonical NF-κB pathway impairs recruitment of RelA and histone demethylase JMJD2A to *Ifnb* promoter, which results in a reduction of H3K4Me3 and H3Ac levels.^372^ These studies reveal the new functions of the non-canonical NF-κB pathway in the anti-virus innate immunity.

### The function of Non-canonical NF-κB in lymphoid organs development

SLOs, such as LNs, PPs, and spleen, provide the optimized microenvironment for immune response.^375^ The three-dimension network of FDC and FRC (fibroblastic reticular cell) inside of lymphoid organ facilities interaction between different types of immune cells to initiate and maintain the appropriate immune response.^307,376^ The development of lymphoid organs occurs during embryogenesis and at the early postnatal stages, which is antigen or pathogen-recognition independent, and highlights the crucial roles of coordinated interactions between hematopoietic cells and stromal cells in SLO organogenesis.^20^ Newly emerging hematopoietic cells are attracted and activated for proliferation and differentiation by homeostatic chemokines, cytokines and growth factors to potential sites of lymphoid organ development. The cytokines produced by hematopoietic cells also induce differentiation and maturation of stromal and endothelial cells, forming the scaffolding of SLO.^377,378^

**Fig. 3:**
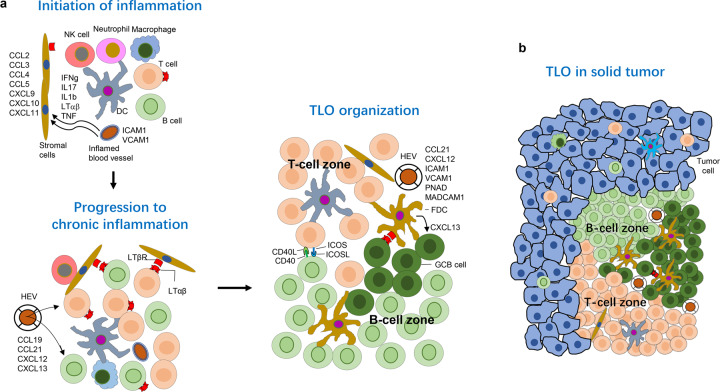
The function of non-canonical NF-κB in lymphoid tissue development and tumor immunology. **a** The inflammatory microenvironment provides the initial signal for TLOs neogenesis. At sites with inflammation, initiated by various innate immune cells (such as macrophages and DCs), various chemokines and cytokines are produced, leading to the recruitment of lymphocytes. The local high endothelial venule (HEV) secrets adhesion molecules VACAM-1, ICAM-1, and MadCAM-1. The lymphocytes interact with local stromal cells, particularly through LTα1β2 and its receptor LTβR, which induces the expression of various chemokines, such as CXCL12, CXCL13, CCL19, and CCL21. Together, these chemokines and adhesion molecules, as well as local stromal cells and FDCs, recruit lymphocytes from nearby HEVs and govern their organization into T-cell and B-cell zones that contain GC. **b** The schematic diagram shows a TLO located within a solid tumor with a T cell zone containing mature DCs and FRCs as well as a B cell zone with GC-B cells and FDCs. DCs, dendritic cells; TLOs, tertiary lymphoid organs; GC, germinal center; FDCs, follicular DCs; FRC, fibroblastic reticular cells

Lymphotoxin-mediated non-canonical NF-κB signal is required for maturation of mesenchymal and stromal cells, and for expression of adhesion molecules and chemokines that determine final structure and function of SLO (Fig. 3). Hematopoietic cells expressing LTα1β2 are identified as LTi (lymphoid tissue inducer) cells, meanwhile stromal cells with LTβR responding to LTα1β2, function as LTo (lymphoid tissue organizer) cells. It is generally accepted that NF-κB is the essential downstream signaling pathway of LTβR for the early expression of adhesion molecules and chemokines by LTo cells, such as CXCL13, CCL21, CCL19 (ELC), and MadCAM-1, all of which are required for LTi recruitment and T- and B-cells migration. LTα KO mice lack all LNs, PPs and suffer from severely disorganized NALTs (nasal-associated lymphoid tissues) and spleen, while LTβ deficient mice lack PLN but retain MLN, sacral and cervical LNs.^379^ However, the phenotype of LTβR KO mice is much more similar to that of LTα KO mice, suggesting that LTα3-activated signal is also functional in certain LN development.^380–383^ RANKL, and its receptor RANK, also participate in SLO development.^384,385^ As mentioned above, the *Aly/Aly* mice, NIK KO mice and IKKα KO mice share a similar phenotype with LTα KO mice in regard to the deficiency of SLO development.^271,311^

TLOs, also known as TLTs (tertiary lymphoid tissues), are the ectopic accumulation of lymphoid cells at sites of persistent infection or chronic inflammation, through lymphoid neogenesis (or lymphoid neo-organogenesis).^386^ Emerging evidence indicates that these unique lymphoid structures are the key players of the local immune response, which could be the novel therapeutic target for cancer, autoimmune and chronic inflammatory diseases.^25^ The major difference between TLO and SLO is that TLOs develop in random, typically non-lymphoid locations in the adults. By contrast, SLOs develop in specified locations at the early stages of life.^387^ However, the mechanisms governing the development of two types of lymphoid organs are very similar. Lymphoid neogenesis can be induced by a variety of stimuli or inhibited by blocking LTβR-Ig proteins, suggesting the essential role of LTβR-mediated NF-κB signaling in TLO development (Fig. 3).

### Non-canonical NF-κB in tumor immunology

Clinical studies indicate the presence of TLOs in microenvironments of colorectal, non-small-cell lung, and breast cancers and melanoma. TLO presence is correlated with a better prognosis.^25,387^ Given the close interaction of tumorigenesis and inflammation, the inflammatory condition in the tumor site provides the suitable microenvironment for TLO neogenesis. As mentioned above, LTβR-mediated non-canonical NF-κB promotes the TLO formation by inducing the expression of adhesion molecules and chemokines such as CXCL12, CXCL13, CCL19 and CCL21 to recruit immune cells (Fig. 3).^388,389^ TLOs modulate the local anti-cancer immune response by recruiting and regulating immune cells, including T cells, B cells, DCs and other myeloid cells. The intratumoral T- and B-cells could be activated to launch antitumor response, as effector and memory cells, with the support of the organized lymphoid architecture in TLO. FRCs, specialized stromal cells localized in the T-cell zone of SLO and TLO, provide structure support, and regulate T-cells and DCs migration and survival.^307,376^ Meanwhile, FDCs in GC participate in organization of lymphoid structure and GC reaction that leads to plasma and memory B-cell production.^25,320,323^ The lymphotoxin-LIGHT signaling provides critical signals for the reticular network development of FRC and FDC.^25^ TLO also facilities T-B cell interaction in which non-canonical NF-κB plays important roles. CD40L-CD40 is critical for T-cell dependent B-cell activation; meanwhile, ICOSL on B-cell functions on T-cell through ICOS to promote Tfh development and GC formation.^3,387,390^ Together with the fact that TLO serves as a hub for the major local antitumor immune response, induction of TLO formation could be a good strategy for tumor immunotherapy.^25,387^ Indeed, it has been reported that the induced intratumoral TLO through injection of LIGHT-VTP (vascular targeting peptide) in solid tumors enhances antitumor immune response.^391^

Hyperactivity of non-canonical NF-κB is associated with malignancies such as multiple myeloma and lymphoma.^392–395^ Mutation of upstream signaling molecules TRAF3 or cIAP leads to abnormal accumulation of NIK and activation of non-canonical NF-κB, resulting in multiple myeloma. On the other hand, deletion of NIK suppresses tumor formation in mouse model.^392^ Besides, rearrangement or mutations of *NF-κB2* have also been found in various human malignancies, including multiple myeloma, T-cell and B-cell lymphoma, with constitutive processing of p100, resulting in sequential non-canonical NF-κB activation.^396–398^ Understanding of these mechanisms is subjected to provide crucial information for discovery of new therapeutic targets for direct suppression of tumorigenesis and tumor growth, as well as to optimize tumor immunotherapy by inducing TLO formation.

## Concluding remarks

Our understating of NF-κB has progressed significantly over the past decade. We now appreciate a crucial and paradoxical function of NF-κB in the regulation of immune response. Gene-editing mouse models, especially conditional deficient mouse models, have allowed us to define the physiological roles of NF-κB in different types of cells and tissues. These models have been particularly important to study this pathway since NF-κB functions in the hematologic cells as well as non-hematologic stromal cells. NF-κB pathway plays pivotal roles in immune homeostasis and chronic inflammation, especially autoimmune diseases, tumorigenesis, chronic inflammatory diseases and aging. More and more efforts have been put into determine how NF-κB functions in autoimmune disease and tumorigenesis, which suggests that this pathway is potentially promising therapeutic target in many diseases. Although there are some NF-κB inhibitors applying in cancer therapy, the efficiency, specificity and side-effects are still significant problems to be solved. Considering the relationship between inflammation and cancer, prevention is a much better way to fight against cancer rather than therapy.
